# Image-free handheld robotic-assisted technology improved the accuracy of implant positioning compared to conventional instrumentation in patients undergoing simultaneous bilateral total knee arthroplasty, without additional benefits in improvement of clinical outcomes

**DOI:** 10.1007/s00167-023-07523-8

**Published:** 2023-08-10

**Authors:** Ali Albelooshi, Muhieddine Hamie, Peter Bollars, Saeed Althani, Rami Salameh, Malak Almasri, Martijn G. M. Schotanus, Prashant Meshram

**Affiliations:** 1 Department of Orthopaedic Surgery, Mediclinic City Hospital, Dubai Healthcare City, Dubai, United Arab Emirates; 2 Orthocure Medical Center, Dubai, United Arab Emirates; 3 Department of Orthopedic Surgery, St. Trudo Hospital, Sint Truiden, Belgium; 4 Department of Orthopedic Surgery, Zuyderland Medical Center, Sittard-Geleen, The Netherlands; 5 School of Care and Public Health Research Institute, Faculty of Health, Medicine and Life Sciences, Maastricht University, Maastricht, The Netherlands

**Keywords:** Bilateral, Simultaneous, Total knee arthroplasty, Robotic, Outcomes, Radiological, Clinical, Functional, Alignment, Satisfaction, TKA, Complication, Revision

## Abstract

**Purpose:**

The purpose of this study was to compare the clinical and radiological outcomes in patients who underwent simultaneous bilateral total knee arthroplasty (SB-TKA) using either robotic-assisted TKA (RA-TKA) or conventional TKA (C-TKA).

**Methods:**

Included were the patients who underwent SB-TKA between January 2018 and January 2020 and had a minimum follow-up of 2 years. Of 151 patients included, 117 patients were operated using an image-free handheld robotic sculpting system (RA-TKA group) and 34 patients operated using conventional instrumentation (C-TKA group). The key outcomes noted were multiple patient-reported outcomes (PROs), adverse events, and radiological outcomes. Two investigators independently measured the radiological outcomes on pre- and post-operative radiographs in coronal plane (medial proximal tibial angle [MPTA] and anatomic lateral distal femoral angle [aLDFA]) and sagittal plane (posterior tibial slope [PTS] and posterior condylar offset [PCO]). The chi-square test was used to examine categorical variables. Student’s t test was used to analyze the continuous variables.

**Results:**

Patients in both groups were similar in baseline characteristics (gender, body mass index, incidence of comorbidities, and length of hospital stay) except that RA-TKA group patients younger (66.7 ± 8.9 vs 70.4 ± 10.5, *P = *0.037) than C-TKA group. The operative time was longer in RA-TKA group as compared to C-TKA (189.3 ± 37.1 vs 175.0 ± 28.2, *P = *0.040). The final PROs at each were similar between the two groups (*P > *0.05). The values of PROs at final follow-up in RA-TKA compared to C-TKA were VAS pain (0.4 ± 0.9 vs 0.4 ± 0.5), KOOS-JR (89.3 ± 5.8 vs 87.1 ± 5.3), and physical (55.9 ± 2.8 vs 55.4 ± 3.2), mental (61.1 ± 4.4 vs 60.2 ± 4.7) component of VR-12 scores, and KSS satisfaction (37.5 ± 1.1 vs 37.1 ± 2.2) (all *P > *0.50 or non-significant [n.s.]). While one patient in RA-TKA required revision of femoral component for peri-prosthetic fracture, none of the patient in conventional group were revised (0.85% vs 0%, *P = *n.s.). The proportion of patients with outliers in RA-TKA group was lower for aLDFA (2.6% vs 22.1%, *P < *0.01) and PTS (0% vs 35%, *P < *0.01).

**Conclusion:**

This comparative study in patients undergoing SB-TKA found reduction of outliers in femoral and tibial implant positioning with RA-TKA as compared to C-TKA. There were no differences in both groups for pain, function, and satisfaction at a minimum of 2 years of follow-up.

**Level of evidence:**

III Therapeutic Study.

## Introduction

Robotic technology use in total knee arthroplasty (TKA) has been introduced to improve the accuracy of implant positioning and achieving target limb alignment in order to reduce residual pain, improve patient satisfaction, and implant survivorship [9]. Previous studies on robotic-assisted TKA (RA-TKA) reported improved accuracy in achieving planned coronal alignment, joint line restoration, and posterior tibial slope (PTS) as compared to conventional TKA (C-TKA) using intramedullary instruments for bone cuts [17]. Despite this improvement in accuracy of implant positioning, RA-TKA has not shown improvement in patient-reported outcomes (PROs) and implant survivorship over C-TKA [12, 21]. There are also concerns of associated learning curve and time and cost efficiency related to the use of RA-TKA [19].

Previous study on bilateral TKA has shown that during simultaneous bilateral TKA (SB-TKA), the potential for errors in accuracy of implant positioning in second knee is 2.5 times as compared to the second knee done as a staged procedure [8]. While several studies have evaluated the efficacy of clinical and radiological outcomes of unilateral or staged bilateral RA-TKA, to the best of our knowledge, only two previous studies have been published so far on simultaneous bilateral TKA (SB-TKA) with an early follow-up of 6 months [2] and 1 year [28] and none at a minimum two years of follow-up. The aim of this study was to compare the clinical and radiological outcomes in patients who underwent SB-TKA using either RA-TKA or conventional instrumentation at a minimum of two years of follow-up. A secondary aim was to compare the radiological outcomes between first operated and second operated knee in SB-TKA patients using either RA-TKA or conventional system. The hypothesis of this study was that in patients undergoing SB-TKA, RA-TKA would improve the accuracy of implant positioning over C-TKA without additional benefits in clinical outcomes.

## Materials and methods

The ethical approval was obtained by the Independent Review Board (Dubai Healthcare City Authority-Regulatory, Dubai, The United Arab Emirates; IRB No. DSREC-12/2022_14) and was conducted according to the principles of the Declaration of Helsinki (2013) and in accordance with the Medical Research Involving Human Subjects Act. Informed consent was obtained from all patients included in the study.

This retrospective single-surgeon case series of 151 patients who had undergone SB-TKA for severe osteoarthritis (Kellgren–Lawrence grade IV on the radiograph [11]) of both knees was between January 2018 and January 2020 at a tertiary care center (Mediclinic City Hospital, Dubai Healthcare City, The United Arab Emirates). The selection criteria of senior surgeon for SB-TKA included patients without a history of cardiovascular disease, blood levels of hemoglobin A1C less than 8%, blood hemoglobin level more than 13 g/dL, 2-dimensional echocardiogram showing normal ejection fraction and no valvular disease, and no history of chronic renal failure. The exclusion criteria were patients with a diagnosis other than primary osteoarthritis (*N* = 0), those with incomplete patient records (*N* = 0), and those who did not complete a follow-up of two years after surgery (*N* = 0). Of 151 SB-TKA patients included in the study period, 117 patients were operated using of an image-free handheld robotic sculpting system (Robot-group; NAVIO Surgical System, Smith & Nephew, Memphis, USA) (RA-TKA group) and 34 were operated using conventional intramedullary rod-based jig instrumentation (C-TKA group). All patients were operated by one senior surgeon (AA) who performs a minimum of 350 TKA procedures annually and using same posterior stabilized TKA system (Anthem, Smith & Nephew, LLC, UK). There were no selection criteria to decide whether the surgery would be done using robotic system or conventional instrumentation. The availability of NAVIO robotic system is difficult to ensure in each primary TKA case as there are only few numbers of this system available in our region. If on the day of surgery, robotic system was not available due to logistical reasons, conventional surgery was done.

### Operative technique

All patients were given a combination of spinal and epidural anesthesia. Tourniquet was not used in any of the patients. A standard midline incision with a medial parapatellar arthrotomy was performed, and exposure of the femur and tibia was carried out with patella eversion. The anterior and posterior cruciate ligaments were sacrificed in all patients. The surgical technique for RA-TKA was same as previously described [4]. The CA-TKA was performed using standard technique using jigs and intramedullary rods. All patients received a combined intravenous and intraarticular routes of tranexamic acid administration. One tranexamic acid dose was given as 15 mg/kg intravenously 10 min before starting the procedure and another dose of 15 mg/kg was given intra-articularly in each knee after capsular closure [1].

In the RA-TKA, the target coronal alignment of knee was based on patient’s preoperative coronal alignment using constitutional alignment philosophy [3]. The target post-operative alignment was an overall 3° varus, wherein 2° varus was in the femur, and 1° varus was in the tibia. The target posterior slope was 3°. In both groups, posterior reference was used for femoral cuts. The post-operative posterior condylar offset (PCO) was targeted as neutral when compared to the preoperative PCO.

### Outcomes

The variables noted in this study included baseline variables, perioperative, and two-year post-operative data. The baseline variables included were age, gender, body mass index (BMI), comorbidities, and blood hemoglobin level at preoperative and 3 days post-operative stage. The operative time was noted in minutes from incision to wound closure per limb. The length of hospital stay was noted in days from hospital admission to discharge. The post-operative outcomes included PROs, adverse events, and radiological results. The PROs and adverse events in post-operative period were the primary outcomes of this study, whereas the radiological findings were the secondary outcomes.

The patients were asked to complete PROs preoperatively and at 3, 6, 12, and 24 months of post-operative follow-ups. The PROs evaluated were the Knee injury and Osteoarthritis Outcome Score for Joint Replacement (KOOS-JR) [14], the Veterans RAND-12 Item Health Survey (VR-12) [26], 10-point visual analog scale for pain (VAS pain), and satisfaction component of knee society score (KSS satisfaction) [29]. The results of the VR-12 are summarized as two scores—a mental component score (VR-12 MCS) and a physical component score (VR-12 PCS). As a routine practice, VR-12 scores were not obtained at 3 months of follow-up because the improvement in general health of patients would not seem to change substantially at 3 months postoperatively and also to reduce the respondents’ burden with multiple PROs in the early post-operative status. The adverse events noted in post-operative period included superficial or deep infection, aseptic loosening, peri-prosthetic fracture, neurovascular injury, complications in RA-TKA group of infection and fracture at site of pin tract used for positioning the robotic tracker, reoperations, and revision defined as change in any or all of prosthesis components.

The plain radiographs of both knees were routinely done while weight-bearing and included anteroposterior and lateral views. Radiographic measurements were made by two arthroplasty fellowship-trained orthopedic surgeons (MH & PM) using a calibrated protocol on digital images using the Impax system for Windows (AGFA HealthCare, Mortsel, Belgium) having accuracy of measurement within 0.1° and 0.1 mm. The radiographs were assessed for variables to assess accuracy in femoral and tibial components positioning in coronal and sagittal planes. For femoral component alignment in coronal plane, the anatomic lateral distal femoral angle (aLDFA) was defined by the angle between the femoral anatomical axis and the articular surface of the distal femur. For tibial component alignment in coronal plane, the medial proximal tibial angle (MPTA) was the medial angle between two lines: one line of the tibial anatomical axis and a second line extending from the medial to the lateral most area of the tibial plateau, while excluding the osteophyte at the tibial plateau surface. The radiological measurements of the tibia component in the sagittal plane, with respect to the anatomical axis, were performed on standard lateral radiographs. The posterior slope of the tibial component (PTS) was defined as the angle on lateral view of knee radiograph between the line perpendicular to the tangent drawn from anterior cortex of the proximal tibia and the tangent line of the tibial plateau. The proportion of patients who had over 3° deviation from target aLDFA of 83°, MPTA of 90°, and PTS of 3° were considered outliers. The PCO was evaluated according to Malviya et al. on lateral radiographs by measuring the maximum distance between the tangent of the posterior cortex of femoral diaphysis and the posterior condylar margin [16]. An outlier in PCO was considered if the difference between preoperative and post-operative values was either ≤ 0.5 mm, 0.6–1.0 mm, or 1.1–1.5 mm.

### Statistical analysis

The analyses were performed with the use of SPSS version 24.0 software (SPSS Inc., Chicago, USA). The power of this retrospective study was determined using post hoc analysis. The effect size for post hoc analysis was calculated as the standardized difference between two means divided by the standard deviation of both groups for the primary PRO of the KOOS-JR [10]. The effect size of this study was determined as 0.4 (moderate) and post hoc power analysis revealed a power of 0.524 (1 − β). The Shapiro–Wilk test showed that the data were normally distributed. The chi-square test was used to examine categorical variables. The statistically significant interactions between both groups were analyzed with Student’s t tests. The inter-reader reliability was determined by comparing radiological measurements between two observers (MH, PM) using intra-class correlation coefficients (ICCs) evaluated by kappa statistics. The intra-reader reliability was determined by comparing the repeated radiological measurements by one of the investigators (MH) done after 5 months of first measurements. The ICC results for reliability were classified, according to Winer et al. [31], as absent to poor (0 to 0.24), low (0.25 to 0.49), fair to moderate (0.50 to 0.69), good (0.70 to 0.89), or excellent (0.90 to 1.0). A subgroup analysis was done within RA-TKA and C-TKA groups to compare the radiological outcomes between first operated and second operated knees. For all analyses, a threshold for all statistical comparisons of *p* value ≤ 0.05 was considered to be statistically significant. The data are presented as either with mean with a SD, 95% confidence interval (95% CI) or proportion (%). If *P* value was > 0.05, it was reported as nonsignificant (n.s.).

## Results

Patients in RA-TKA group were younger than C-TKA group (Table 1). The operative time was longer in RA-TKA group. Preoperatively, patients in both groups were not significantly different for all PROs of KOOS-JR, VR-12 physical. VR-12 mental, and VAS pain (Table 2). The mean PROs at 3, 6, 12, and 24 months of follow-up and mean KSS satisfaction at 1  and 2 years of follow-up were similar between two groups (*P = *n.s.).

**Table 1:** Patient demographics and perioperative variables of patients undergoing simultaneous bilateral total knee arthroplasty in RA-TKA and C-TKA groups

	RA-TKA group (*n* = 117)	C-TKA group (*n* = 34)	*P* value
Age at index surgery, years	66.7 (8.9)	70.4 (10.5)	.037
Sex, male (%)	29 (24.8)	11 (32.4)	n.s.
BMI, kg/m^2^	33.4 (6.0)	32.2 (5.4)	n.s.
Incidence of Comorbidities			
Diabetes mellitus (%)	52(44)	20(59)	n.s.
Chronic obstructive pulmonary disease (%)	23(20)	11(32)	n.s.
Ischemic heart disease (%)	36(31)	9(26)	n.s.
Hypertension (%)	57(49)	18(53)	n.s.
Mean operative time, minutes (SD)	189.3(37.1)	175.0(28.2)	.040
Mean difference in hemoglobin level from post-operative day 0 to day 3, gm/dL (SD)	3.2 (1.4)	3.3 (1.2)	n.s.
Mean length of hospital stay, days (SD)	4.3 (0.7)	4.8 (1.5)	n.s.

*RA-TKA* robotic-assisted total knee arthroplasty, *C-TKA* conventional total knee arthroplasty, *SD* standard deviation; n.s. nonsignificant indicating a *P* value > 0.05

**Table 2:** Comparison of patient-reported outcomes at preoperative and post-operative 3, 6, 12, and 24 months of follow-up between RA-TKA and C-TKA groups

Patient reported outcome	Time	RA-TKA (*N* = 117)			CA-TKA (*N* = 34)			*P* value
		Mean	SD	(95% CI)	Mean	SD	(95% CI)	
KOOS-JR	Preoperative	31.3	7.3	30.0–32.7	29.1	10.7	25.4–32.8	n.s.
	3 months	80.3	8.3	78.8–81.9	78.1	8.3	75–81.2	n.s.
	6 months	84.9	10.5	83.0–86.8	85.4	8.2	82.5–88.2	n.s.
	12 months	88.0	7.9	86.5–89.4	89.2	8.6	86.2–92.2	n.s.
	24 months	89.3	5.8	88.3–90.4	87.1	5.3	85.3–89	n.s.
VR-12–Physical	Preoperative	25.1	2.9	24.6–25.6	25.3	3.1	24.1–26.3	n.s.
	6 months	49.8	4.5	49–50.6	49.5	4.3	48.7– 50.2	n.s.
	12 months	54.4	3.8	53.7–55.1	53.6	3.5	52.4–54.8	n.s.
	24 months	55.9	2.8	55.4–56.4	55.4	3.2	54.3–56.5	n.s.
VR-12–Mental	Preoperative	33.6	9.2	31.9–35.3	33.3	8.4	30.3–36.2	n.s.
	6 months	55.3	4.6	54.4–56.1	55.5	4.4	54–57	n.s.
	12 months	59.3	4.4	58.5–60.1	60.9	3.3	59.7–62	n.s.
	24 months	61.1	4.4	60.3–61.9	60.2	4.7	58.6–61.9	n.s.
VAS–Pain	Preoperative	9.4	0.6	9.3–9.5	9.4	0.6	9.2–9.6	n.s.
	3 months	2.9	1.1	2.7–3.1	3.4	0.9	2.2–3.3	.018
	6 months	1.9	1.0	1.7–2.1	2.2	1.0	1.9–2.5	n.s.
	12 months	1.2	0.8	1.1–1.6	1.1	1.4	10.9– 1.9	n.s.
	24 months	0.4	0.9	0.2–0.5	0.4	0.5	0.2–0.6	n.s.
Satisfaction KSS	12 months	37.2	3	36.7–37.8	36.9	4.6	35.3–38.5	n.s.
	24 months	37.5	1.1	37.3–37.7	37.1	2.2	36.3–37.9	n.s.

*RA-TKA* robotic-assisted total knee arthroplasty, *C-TKA* conventional total knee arthroplasty, *SD* standard deviation, *KOOS-JR* Knee injury and Osteoarthritis Outcome Score for Joint Replacement, *VR-12* Veterans RAND-12 Item Health Survey, *VAS* visual analog scale, *KSS* knee society score, n.s. nonsignificant indicating a *P* value > 0.05

The rate of revision was not different between RA-TKA and C-TKA groups (0.85% vs 0%, *P = *n.s.). In RA-TKA group, one patient required revision and one required reoperation without replacing any of the prosthesis components. First patient suffered from post-traumatic peri-prosthetic femur fracture in the first operated knee after one month of index SB-TKA requiring revision with cone and constrained polyethylene. Another patient in RA-TKA group had persistent pain in the knee, operated second in sequence, due to prominent cement of 10 × 5 mm in lateral distal femoral condyle causing soft tissue impingement. At 2 months of follow-up, this patient underwent removal of the prominent cement using a small incision.

In C-TKA group, none of the patients required revision surgery. Three patients required reoperation in the knee operated second in sequence during SB-TKA. One patient had lateral subluxation of the patella and anterior knee pain requiring arthroscopic release of lateral retinaculum. Two other patients had anterior knee pain in post-operative period and underwent patella resurfacing at 12 months of follow-up. None of the patients had other complications, such as infection, neurovascular injury, or aseptic loosening of the components.

The inter-observer and intra observer reliability of radiological measurements was good to excellent (Table 3). The results of accuracy of component positioning showed less proportion of outliers in RA-TKA group for aLDFA and PTS than C-TKA group (Table 4). The subgroup analysis comparing the radiological outcomes of first operated knee with second operated knee did not show any difference in both the groups (Table 5).

**Table 3:** The inter- and intra-reader reliability of each radiological measurement was assessed using ICC by kappa statistics

	First reader measurements	Repeated measurements by first reader	Second reader measurements	Inter-reader ICC	Intra-reader ICC
aLDFA, degrees	83.5 (2.2)	83.4 (2.2)	83.2 (2.4)	0.917	0.952
MPTA, degrees	86.2 (3.7)	86.5 (3.0)	86.2 (4.1)	0.888	0.965
PTS, degrees	9.9 (5.1)	12.6 (6.8)	8.5 (4.9)	0.906	0.994
PCO, mm	3.3 (0.4)	3.5 (0.5)	3.3 (0.4)	0.801	0.836

All values are expressed in mean and standard deviation

*ICC* intra-class correlation coefficient, *CI* confidence interval, *RA-TKA* robotic-assisted total knee arthroplasty, *C-TKA* conventional total knee arthroplasty, *Sd* standard deviation, *MPTA* medial proximal tibial angle, *SD* standard deviation, *aLDFA* anatomic lateral distal femoral angle, *PTS* posterior tibial slope, *PCO* posterior condylar offset

**Table 4:** Postoperative radiographic outcome of the accuracy of femoral and tibial components placement between RA-TKA and C-TKA groups

	RA-TKA (*N* = 234)	C-TKA (*N* = 68)	*P* value
aLDFA, degrees			
Mean (SD)	83.7 (1.2)	84.0 (2.3)	n.s.
Range min–max	80–88	79–88	
Target	83	83	
Number of outliers (%)	6 (2.6)	15 (22.1)	.000
MPTA, degrees			
Mean (SD)	89.7 (0.8)	89.3 (1.1)	.000
Range min–max	87–91	86–92	
Target	90	90	
Number of outliers (%)	0 (0)	1 (1.5)	n.s.
PTS, degrees			
Mean (SD)	3.5 (0.6)	5.8 (1.5)	.000
Range min–max	2–5	2–8	
Target	3	3	
Number of outliers (%)	0 (0)	24 (35.3)	.000
PCO, mm			
*Mean (SD)	3.2 (0.4)	3.2 (0.4)	n.s.
Ratio (SD)	0.3 (0.2)	0.3 (0.2)	n.s.
≤ 0.5 mm (%)	199 (85)	58 (85)	
0.6–1.0 mm (%)	31 (13)	9 (13)	
1.1–1.5 mm (%)	4 (2)	1 (2)	n.s.

*RA-TKA* robotic-assisted total knee arthroplasty, *C-TKA* conventional total knee arthroplasty, *SD* standard deviation, *MPTA* medial proximal tibial angle, *SD* standard deviation, *aLDFA* anatomic lateral distal femoral angle, *PTS* posterior tibial slope, *PCO* posterior condylar offset, *n.s.* nonsignificant indicating a *P* value > 0.05

**Table 5:** Postoperative radiographic outcome of the accuracy of femoral and tibial components placement between first and second operated knee in RA-TKA and C-TKA groups

	RA-TKA			C-TKA		
	First operated KNEE (*N* = 117)	Second operated knee (*N* = 117)	*P* value	First operated knee (*N* = 34)	Second operated knee (*N* = 34)	*P* value
aLDFA, degrees	84.0 (2.0)	84.3 (2.3)	n.s.	83.5 (7.6)	84.1 (1.6)	n.s.
MPTA, degrees	89.2 (0.9)	89.4 (1.4)	n.s.	89.4 (1.0)	89.5(1.0)	n.s.
PTS, degrees	6.2 (1.6)	6.3 (1.7)	n.s.	4.6 (1.9)	4.5 (1.8)	n.s.
PCO, mm	3.3 (0.4)	3.3 (0.5)	n.s.	3.3 (0.4)	3.3 (0.4)	n.s.

All values are expressed in mean (standard deviation)

*RA-TKA* robotic-assisted total knee arthroplasty, *C-TKA* conventional total knee arthroplasty, *MPTA* medial proximal tibial angle, *aLDFA* anatomic lateral distal femoral angle, *PTS* posterior tibial slope, *PCO* posterior condylar offset; *n.s.* nonsignificant indicating a *P* value > 0.05

## Discussion

The most important finding of this study was that there was no difference in PROs, satisfaction, and incidence of adverse events at final follow-up between the RA-TKA and C-TKA groups. However, RA-TKA improved accuracy of femoral implant positioning in coronal plane (aLDFA) and tibial implant positioning in sagittal plane (PTS) than CA-TKA.

This is the first study evaluating PROs at a minimum two years of follow-up in patients undergoing SB-TKA either using RA-TKA or CA-TKA.

First key finding of this study was that the PROs of KOOS-JR, VR-12 physical, VR-12- mental, and VAS pain at 3, 6, 12, and 24 months of follow-up and revision rate were not significantly different in RA-TKA and C-TKA groups. The mean patient satisfaction score was also similar in both groups. These findings are similar to other studies on SB-TKA which at 6 months [2] and 1 year [28] of follow-up found no statistical difference in PROs and revision rate with RA-TKA or C-TKA. On the contrary, Zhang et al. in their metanalysis reported statistically significantly better PROs with RA-TKA than C-TKA. However, the difference between the two groups for mean KSS of 1.23 and mean Western Ontario and McMaster Universities Osteoarthritis Index/WOMAC score of 3.72 is lower than the published threshold values of minimally clinically important difference which are 8.9 for KSS and 15 for WOMAC [7, 13, 18]. Thus, the statistically significant improvement in PROs found in Zhang et al. study would not be clinically relevant in patients’ perspective [18].

There have been concerns of several complications associated with RA-TKA tracker pin site including superficial infections, osteomyelitis, stress fracture, vascular injury, hematoma, myositis ossificans, and mechanical failures [17, 20]. While one patient in this study in RA-TKA group did have a peri-prosthetic fracture in first operated knee, it is less likely to be attributed to the pin tract of robotic tracker as the fracture pattern was intraarticular and did not involve pin tract site and was associated with history of trauma. None of the patients in our study had pin-site complications until last follow-up. It is noteworthy that all the three patients that needed reoperation were the second operated knee in SB-TKA sequence and belonged to C-TKA group. There is limited literature to indicate if the second operated knee is at higher risk of complications and reoperation. The influence of robotic use on the occurrence of complications in second operated knee also needs further investigation.

The study result of improved efficacy of implant positioning with RA-TKA as compared to CA-TKA when operated by one senior surgeon with over 15 years’ experience resonates with several previous studies. Song et al. [28] conducted a randomized controlled trial in 30 patients undergoing SB-TKA wherein one knee had RA-TKA using a preoperative CT scan-based robotic assistance (ROBODOC, Integrated Surgical Systems, Sacramento, CA) and second knee had C-TKA. They found similar results to this study of significantly less proportion of outliers with RA-TKA in femoral component positioning in coronal plane and tibial components positioning in sagittal plane. Batallier et al. in their cohort of SB-TKA also reported improved accuracy of femoral component placement with RA-TKA as compared to C-TKA [2]. Zhang et al. [32] conducted a metanalysis of 16 studies published till October 2020 on efficacy of RA-TKA (semi-active robotic systems) over C-TKA and found that RA-TKA statistically significantly improved accuracy of femoral and tibial coronal components and tibial sagittal component positioning as compared to C-TKA. Contrary to the findings of this metanalysis, our study did not find reduction in outliers in PCO in RA-TKA group as compared to C-TKA. Nonetheless, our study findings further strengthen the evidence that RA-TKA improves the accuracy of implant positioning as compared to C-TKA. Follett et al. reported reduced accuracy of implant positioning with C-TKA in second operated knee than first operated knee during SB-TKA [8]. Interestingly, the current study did not find differences in accuracy of implant placement during SB-TKA for the first operated knee compared to the second operated knee in both RA-TKA and C-TKA groups. Further investigations are needed to evaluate whether RA-TKA has advantage over C-TKA of consistent improvement in accuracy of implant placement in first versus second operated knee during SB-TKA.

Another interesting finding of this study that is that RA-TKA took more surgical time than C-TKA similar to previous studies [28, 32]. However, as compared to the study in SB-TKA by Song et al. [28] that used CT-based ROBODOC RA-TKA system and reported a mean operating time over C-TKA of 25 min, the imageless NAVIO RA-TKA system used in this study required lesser surgical time of a mean of 12 mins over C-TKA. The newer second-generation imageless RA-TKA such as CORI could further reduce the surgical time compared to first-generation NAVIO RA-TKA by utilizing high-speed cameras to increase the speed of surgical navigation and high-speed burrs for faster rate of bone removal [4]. Notably, the duration of surgery using RA-TKA is reported to reduce after initial learning curve of 5–43 cases [5]. One of the ways to flatten the learning curve of RA-TKA could be by having a surgeon with robotic system experience present intraoperatively [25]. There are several studies that reported similar operative time between RA-TKA and C-TKA after initial learning curve with the former which is contrary to the findings of this study [5].

The current study used constitutional alignment concept with a wider acceptability of 3 degrees in variation from neutral hip–knee–ankle axis (HKAA), which found no differences in PROs between conventional instrumentation and RA-TKA. The proposed concepts target limb alignment in coronal plane to give optimal TKA results include the mechanical alignment [3], kinematic alignment [22, 24], and functional alignment which is based on the achievement of preoperative joint line obliquity and the Coronal Plane Alignment of the Knee (CPAK) classification category [22, 30]. There are a few studies that showed improved clinical outcomes with TKA performed using functional alignment as compared to mechanical alignment [6, 23]. However, there is no consensus regarding the “ideal” target of coronal limb alignment after TKA which would give result in the best long-term results of functional recovery and longevity of the prosthesis. Long-term prospective studies comparing the efficacy of functional alignment principle with RA-TKA with the traditional mechanical alignment concept for function and longevity are needed in future to understand the “ideal” tools and principle for performing TKA.

The findings of this study should be viewed in light of its limitations. First limitation is the retrospective study design. Second limitation is that the number of patients in C-TKA group (*N* = 34) was lower than those in RA-TKA group (*N* = 117), which could have led to type 2 errors in statistical analysis. It should be noted that the number of patients in C-TKA group is still comparable or more than the previous similar studies [2, 28]. Another limitation of this study is that analysis between first and second operated knee in RA-TKA and C-TKA groups for functional outcomes and recovery time could not be performed due to lack of availability of this data.

## Conclusion

This comparative study in patients undergoing SB-TKA found reduction of outliers in femoral and tibial implant positioning with RA-TKA as compared to conventional instrumentation. Despite improved accuracy, there were no differences in both groups for pain, function, and satisfaction at a minimum of 2 years of follow-up.

## Data Availability

The data that support the findings of this study are not openly available due to reasons of sensitivity and are available from the corresponding author upon reasonable request.
